# Improving PacBio Long Read Accuracy by Short Read Alignment

**DOI:** 10.1371/journal.pone.0046679

**Published:** 2012-10-04

**Authors:** Kin Fai Au, Jason G. Underwood, Lawrence Lee, Wing Hung Wong

**Affiliations:** 1 Department of Statistics, Stanford University, Stanford, California, United States of America; 2 Pacific Biosciences of California, Menlo Park, California, United States of America; University of Iowa, United States of America

## Abstract

The recent development of third generation sequencing (TGS) generates much longer reads than second generation sequencing (SGS) and thus provides a chance to solve problems that are difficult to study through SGS alone. However, higher raw read error rates are an intrinsic drawback in most TGS technologies. Here we present a computational method, LSC, to perform error correction of TGS long reads (LR) by SGS short reads (SR). Aiming to reduce the error rate in homopolymer runs in the main TGS platform, the PacBio® *RS*, LSC applies a homopolymer compression (HC) transformation strategy to increase the sensitivity of SR-LR alignment without scarifying alignment accuracy. We applied LSC to 100,000 PacBio long reads from human brain cerebellum RNA-seq data and 64 million single-end 75 bp reads from human brain RNA-seq data. The results show LSC can correct PacBio long reads to reduce the error rate by more than 3 folds. The improved accuracy greatly benefits many downstream analyses, such as directional gene isoform detection in RNA-seq study. Compared with another hybrid correction tool, LSC can achieve over double the sensitivity and similar specificity.

## Introduction

The advent of second generation sequencing (SGS) has opened up a new era of genome-wide and transcriptome-wide research. Currently a single lane of a SGS instrument such as the HiSeq® instrument from Illumina can generate 10^8^ short reads (SR) of length up to 200 bp with a low error rate (2%) [1]. While the high read count of SGS allows for accurate quantitative analysis, the relatively short length of the reads greatly reduces the utility of SGS in tasks such as de novo genome assembly and full length mRNA isoform reconstruction. Given RNA-seq with SRs only, the reconstruction of gene isoforms must rely on assumptions. For example, SLIDE [2] uses statistical modeling under sparsity assumption, and Cufflinks [3] imposes solution constraints. These assumptions are not supported by direct experimental evidence and may induce many false positives.

Recently, a third generation sequencing (TGS) technology capable of much longer reads has become available. The PacBio *RS* can yield reads of average length over 2,500 bp and some longer reads can reach 10,000 bp [4]. These continuous long reads (CLR) can capture large isoform fragments or even full length isoform transcripts. These CLRs tend to have high error rate (up to 15%). The sequencing accuracy can be greatly improved by approach called “circular consensus sequencing” (CCS) which uses the additional information from multiple passes across to insert to build a higher intramolecular accuracy. However, the requirement that 2 or more full passes across the insert for CCS read generation limits the insert size to <1.5 Kb for Pacific Biosciences’ C2 chemistry and sequencing mode, not allowing the interrogation of extremely long transcripts by CCS reads. Furthermore, the number of reads per run from the PacBio *RS* is in only the range of 50,000 per run. The relatively modest throughput makes it difficult to obtain full sampling of the transcriptome.

Some researchers have been attempting to combine PacBio long reads and SGS short reads, for example the genome assembler Allpaths-LG [5]. In this paper, we introduce a error correction approach that combines the strengths of SGS and TGS for in the task of isoform assembly from RNA-seq data. In particular, we use homopolymer compression (HC) transformation as a means to allow accurate alignment of SR to LR. HC transformation has been previously been proven to be useful in seeking possible alignment matches [6] of pyrosequencing reads (454 platform). Since the SRs have lower sequencing error, the LR can be modified based on information from the aligned SRs to form a “corrected” LR with a much lower error rate than that of the original LR.

This method was implemented in the Python program LSC, which is freely available for the research community and can be downloaded at http://www.stanford.edu/~kinfai/LSC/LSC.html.

## Methods

**Figure 1:**
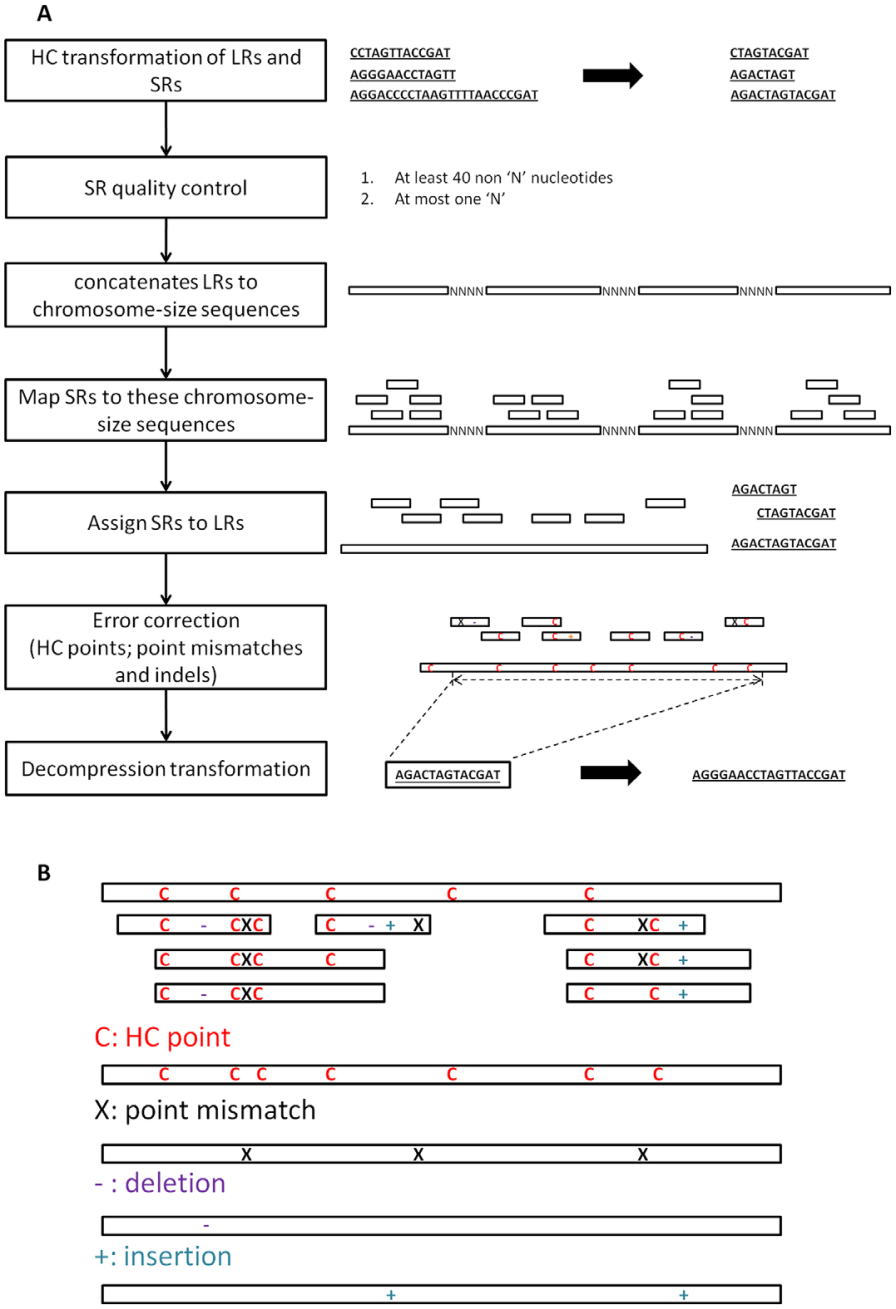
The workflow of standard LSC and the outline of error correction based on HC transformation. (a) LSC consists of five steps: HC transformation of SRs and LRs, SR quality control, SR-LR alignment, error correction and decompression transformation. LSC outputs the sequence from the left-most SR-covered point to the right-most SR-covered one. (b) In the SR-LR layout, correction points consist of four types: HC points, point mismatches, deletions and insertions. Each correction point is treated independently and replaced by the consensus sequence from SRs for the first three types. Insertion sequence at position i is treated as a whole at the gap between two positions i and i+1 of the compressed LRs. A consensus sequence of this gap is inserted to the final output at the corresponding position.

There are five main steps in LSC: HC transformation, SR quality control, SR-LR alignment, error correction and decompression transformation (Figure 1A).

### Homopolymer Compresssion

The sequences in LRs and SRs are transformed by homopolymer compression so that each homopolymer run is replaced by a single nucleotide of the same type, for example, a run of 5 consecutive adenosines, “AAAAA” is replaced by a single “A”. If the original SR is of reasonable length (> = 75 bp), the compressed SR is likely to be still of sufficient length to allow reliable alignment to the compressed LRs. For a human chromosome, the ratio of its HC compressed length to its original length is around 0.65 (Table S1). For example, 75 bp SRs are compressed to ∼48 bp at average. However, in a compressed read, any two consecutive nucleotides must be different, which further reduces the information content useful for alignment. Instead of having 4 degrees of freedom (A,C,G,T) in each new position of the raw reads, compressed reads only possess 3 degrees of freedom. Therefore, in terms of finding an repeat alignment hits by chance, compressed reads have equivalent length of regular reads by a factor log_4_(3). Thus a HC transformed 75 bp SR is roughly equivalent to a regular 40 bp SR in terms of its ability to identify a genomic location. This is generally sufficient unless the SR is from a repetitive region.

To illustrate this with read data, we use 64,313,204 X 75 bp SGS (Illumina) reads as an example, and mapped them (before or after compression) to human RefSeq annotation by Novoalign (V2.07.10) [7] and allowed 1 bp mismatch. 37,564,778 reads are mapped to 70,247,943 hits before compression. 37,556,821 reads are mappable after compression and mapped to 70,468,788 hits. Therefore, with compression, very little sensitivity is sacrificed in mapping (0.02%) and also very little in specificity (0.31%).

**Figure 2:**
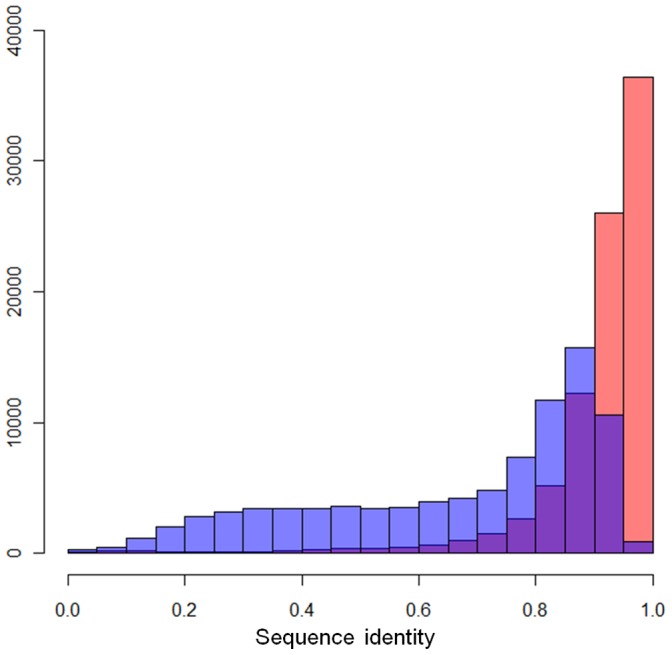
The histogram of sequence identities of ecLRs by LSC and rLRs. Red bars are the LSC ecLRs and purple ones are rLRs. After corrections, much more ecLRs have accuracy higher than 0.9.

**Table 1:** The comparison of the identities between LSC ecLRs and rLRs.

Sequence identity (I)	LSC ecLR	rLR
I < = 0.8	8,485 (9.42%)	51,145 (56.81%)
0.8<I< = 0.9	17,328 (19.25%)	27,431 (30.47%)
0.9<I< = 0.95	26,043 (28.93%)	10,543 (11.71%)
0.95<I< = 1.00	36,355 (40.38%)	875 (0.97%)

**Figure 3:**
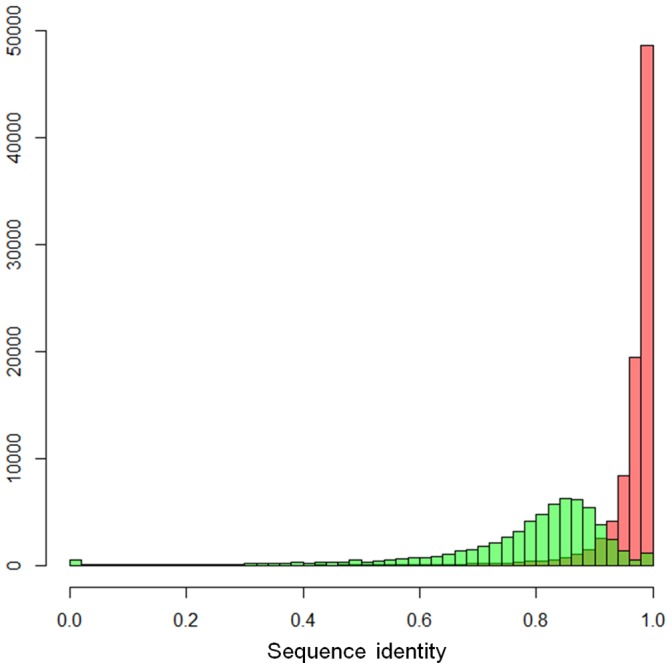
The comparison of sequence identities between SR-covered/SR-uncovered regions in each ecLRs. The sequence identity distribution of SR-covered regions is in red and SR-uncovered one in green.

**Figure 4:**
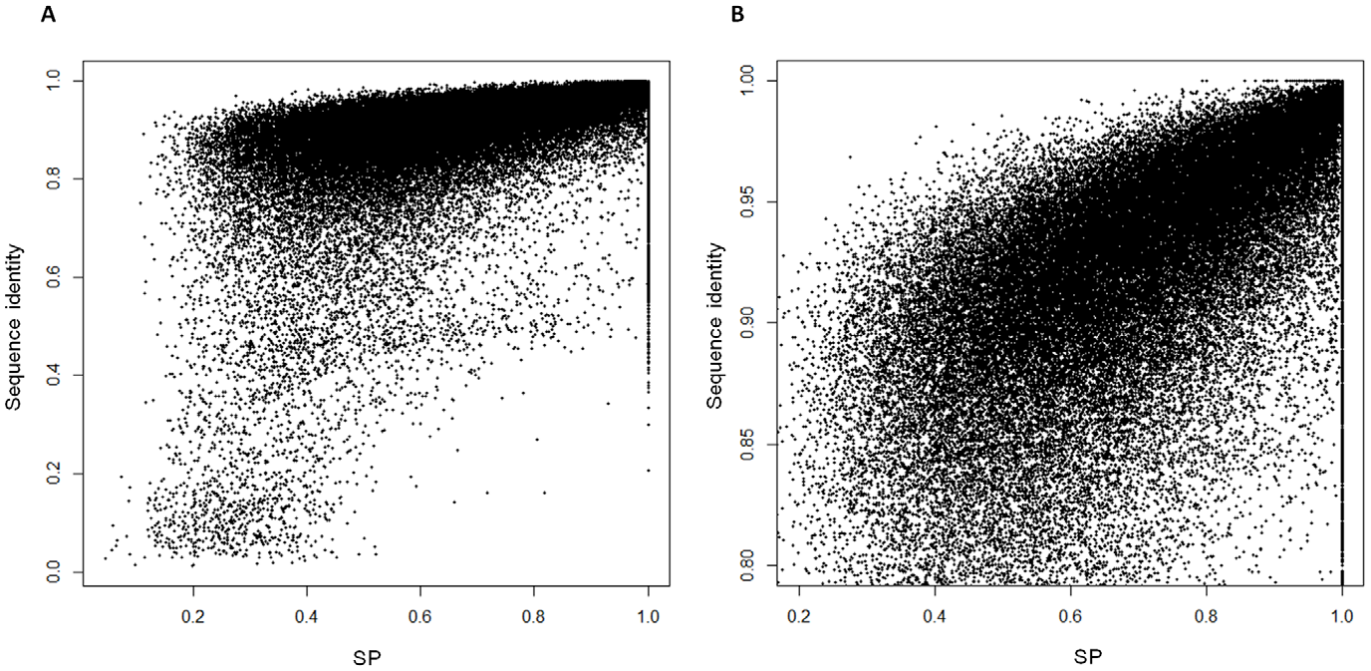
The scatter plots of SR-covered sequence percentage (SP) and sequence identity of ecLRs. (a) overview (b) zoom-in view from SP of 0.2 to 1.0 and sequence identity from 0.8 to 1.0. Sequence identity is positively related with SP.

### SR Quality Control

Some of the compressed SRs are of poor quality either because they are very short or contain uncertain bases (indicated by ‘N’). Such reads are likely to cause alignment errors. LSC filters out compressed SRs that have less than 40 ‘non-N’ nucleotides or have more than one ‘N’ by default. These parameters can be changed in the LSC configuration file according to data quality.

**Table 2:** The percentage of matches with the reference genome (hg19) in different SCD bins.

SCD	The percentage of matches	Number of matches	Number of mismatches
0	0.7250	13,422,373	5,090,948
1	0.9073	5,105,739	521,544
2	0.9413	2,365,912	147,471
3	0.9547	1,512,073	71,607
4	0.9618	1,113,180	44,209
5	0.9659	888,164	31,314
6	0.9708	739,542	22,216
7	0.9723	633,977	18,032
>7	0.9820	20,980,352	385,334

**Figure 5:**
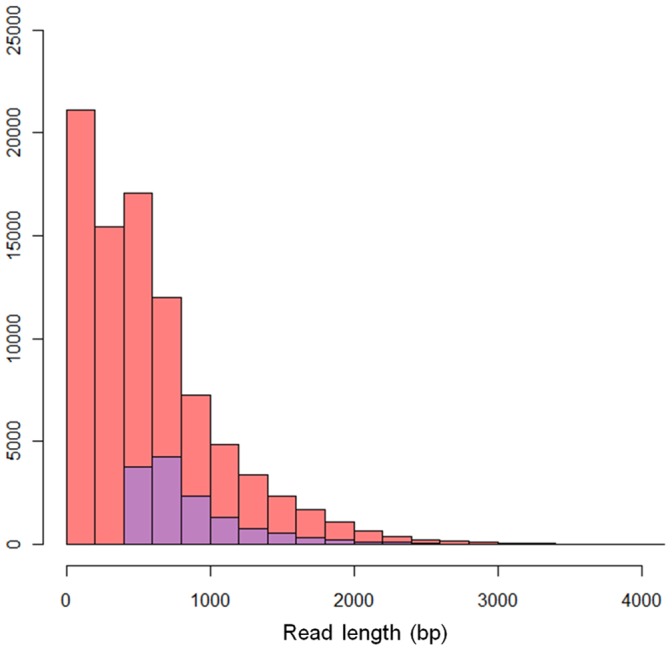
The histogram of lengths of LSC ecLRs (red bars) and PacBioToCA ecLRs (purple bars). There are much more ecLRs from LSC than PacBioToCA in every bin.

**Figure 6:**
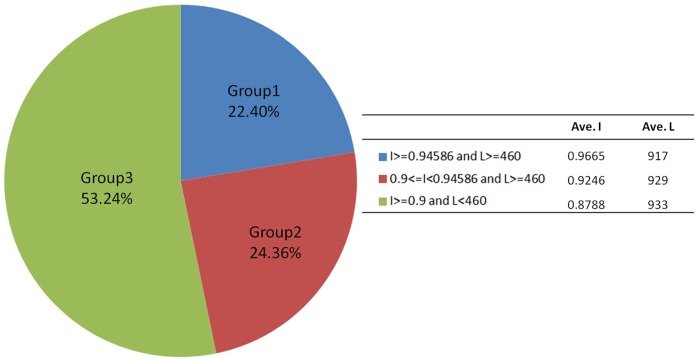
The pie chart of the LSC ecLRs (I> = 0.9). The LSC ecLRs are categorized by their identities and lengths. 22.40% of these outputs are the comparable result with PacBioToCA, while LSC also output many other ecLRs with good accuracy with various lengths.

**Table 3:** The comparison of the overall performance of LSC and PacBioToCA.

	LSC	PacBioToCA
**Running time**	10 hours (8 hours Novoalign+2 hours LSC)	81 hours
**Hard disk usage**	20 G	800 ∼ 1,000 G
**Output**	Top 13,995 ecLRs (> = 460 bp)*	13,995 ecLRs
**Averaged length**	917 bp	880 bp
**Averaged I**	0.9664	0.9603

* Among all LSC ecLRs longer than or equal to 460 bp, the best 13,995 outputs have sequence identities I> = 0.94586.

**Table 4:** The numbers of reads that cover different numbers of known splices in Ensembl.

		Group 1	Group 2
Covering all splices of a transcript*		4,181	3,819
**Covering partial transcript**	**# covering splices = 0 (exonic reads)**	2,247	1,896
	**# covering splices = 1**	1,204	1,693
	**# covering splices = 2**	1,543	1,987
	**# covering splices = 3**	1,269	1,636
	**# covering splices = 4**	1,150	1,277
	**# covering splices = 5**	731	914
	**# covering splices >5**	1,670	1,968
**Total**		13,995	15,190

* The read cover all splices of a transcript.

**Figure 7:**
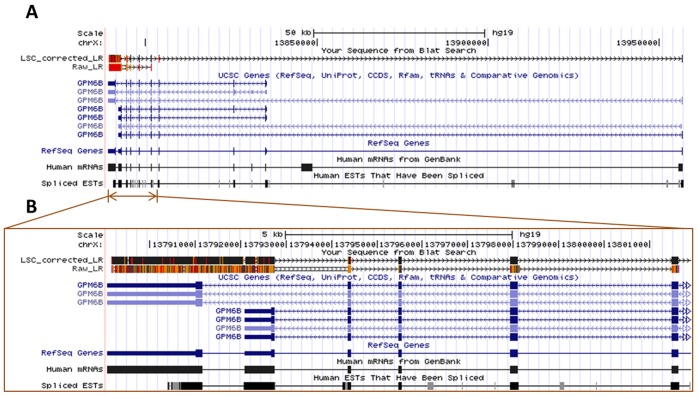
The overview and the zoom-in view of a new 3′ UTR isoform of GPM6B detected by an LSC ecLR (4,259 bp). (a) without error correction by LSC, the rLR cannot detect two 3′ end exons of this isoform because of the high error rate. GPM6B encodes a membrane glycoprotein that belongs to the proteolipid protein family. Proteolipid protein family members are expressed in most brain regions and different isoforms of GPM6B could alter cell-to-cell communication. (b) after correction, there are much less errors (marked in orange and red) in the exons.

### SR-LR Alignment

The basic assumption of LR error correction is that the aligned SRs and LRs are derived from the same source in the source sequence. In genome sequencing, the source sequence is a segment of genomic sequence, whereas in RNA-seq, it is a segment of a cDNA. In either case, SRs can be mapped to LRs with only allowance for substitutions or small indels. This is in contrast to standard RNA-seq alignment to the reference genome which must allow for large introns. In this work, we use Novoalign (V2.07.10) as the default aligner. Novoalign is known to have high sensitivity and specificity, but is computationally demanding. If computational efficiency is a concern, Novoalign can be replaced by a faster alternative such as BWA [8] or Seqalto [9].

As input to Novoalign, LSC concatenates the HC transformed LRs together into human chromosome-sized reference sequences with n bp poly-N inserts between successive LRs, where n is the length of original SRs. This is regarded as the reference genome against which the HC transformed SRs are aligned.

### Error Correction

After SR alignment, the alignment of each HC transformed LR and the corresponding SRs are laid out as in Figure 1B. The LR is then modified according to consensus information from the aligned SRs in order to correct LR errors. Error correction is performed at four types of correction points: HC points, point mismatches, deletions and insertions. Both HC points in LR and SRs are considered to be potential correction points. At each correction point of the first three types, the sequences of all SRs that cover it are decompressed temporarily and the consensus sequence of them replaces this correction point. For insertion points, at first LSC checks whether the majority of aligned SRs have insertions at this point or not. If yes, the consensus decompressed sequence inserts at this point.

### Decompression

After all correction points are replaced with their SR-consensus sequences, all remaining (i.e. uncorrected by SRs) HC points are decompressed. Lastly, LSC outputs the decompressed sequence from the left-most SR-covered point to the right-most SR-covered point, while the SR-uncovered regions at 3′/5′ ends are not included.

## Results

We tested LSC on two RNA-seq data sets: (1) human brain cerebellum polyA RNA processed to enrich for full-length cDNA for the PacBio *RS* platform under C2 chemistry conditions as CLR data (http://www.stanford.edu/~kinfai/human_cerebellum_PacBioLR.zip, 108,767 reads, median sublength at 718 bp, maximum sublength at 8,259 bp), and (2) human brain data from Illumina’s Human Body Map 2.0 project (GSE30611, 64,313,204 single end reads, 75 bp) as SR data. After applying the default filtering described in the “Methods” section, 63,519,800 SRs were retained. LSC output 90,036 error-corrected LRs (ecLR).

### Comparison between LRs w/o LSC Correction

The segment between the left-most SR-covered point to the right-most SR-covered point in the raw LR is denote as rLR. rLRs were mapped to the human genome (hg19) by BLASR [10], which was developed specifially for PacBio data alignment. ecLRs were mapped to the human genome by BLAT. As a measure of LR accuracy, the sequence identity (I) is defined as:
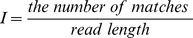



The majority of ecLRs have sequence identity (I) higher than 0.9. In contrast, the sequence identity of rLRs spread from 0.2 to 0.9 (Figure 2). Out of all 90,036 ecLRs, 62,465 (69.31%) have identity higher than or equal to 0.9, while only 11,418 rLRs pass this cutoff (Table 1). This suggests that ecLRs will be much more informative than rLRs in downstream analyses such as assembly of gene isoforms. We note that the identity value of I = 0.9 in ecLRs does not mean that the error rate is about 10% even after correction. The error rate at the SR-covered region within the LR will be much lower, as will be seen below (Table 2). Both 5′ and 3′ ends of ecLRs are SR-covered, so their accuracies are high and allows reliable assembly.

We also compared raw and corrected LRs on their ability to detect exon junctions from the alignment results above. 60,931 junctions are detected from the full-length raw LRs with EST validation rate of 56.28%. In contrast, 206,794 junctions are detected from ecLRs with EST validation rate of 68.18%. Thus, based on EST evidence, ecLR can lead to much higher sensitivity and specificity in exon junction detection.

### Dependence of Correction Efficiency on SR Coverage

LSC outputs the span from the left-most SR-covered position to the right-most SR-covered position, so ecLR also contains positions not covered by any SRs (SR-uncovered regions). These positions will not be edited in any way by LSC and therefore should have an error rate similar to the rLRs. Figure 3 shows significant identity differences between SR-covered and SR-uncovered regions in each reads. The identity distribution of SR-uncovered regions has a beta distribution shape centered at ∼0.85 with wide variance, while most SR-covered regions pile up at high accuracy range (I>0.9) with a small tails from 0.9 to 0.8. Therefore, the overall accuracy of ecLR depends on SR-covered sequence percentage (SP) that is defined as the length of SR-covered region divided by the length of ecLR. The strong positive relationship with SP is shown in Figure 4.

To see how the error rate is reduced by increasing SR coverage, we define the SR-covered depth (SCD) at a position of the ecLR as the number SRs whose alignments overlap that position. All of the nucleotide positions that the 90,036 ecLRs output by LSC are categorized into 9 bins according to their SCD (Table 2). The SR-uncovered positions (SCD  = 0) are not corrected by LSC and only 72.50% of them match the reference genome. However, this percentage increases to 90.73% with SCD  = 1, which suggests a significant reduction of error as soon as here is any SR aligned to that position. As SCD increases, the percentage of matches increases gradually and reaches the plateau of 98% at 8-SCD, which is comparable with the accuracy of SGS. Therefore, with sufficient SR data with sufficient depth, LSC will have an error rate as low as that of SGS.

### Comparison of LSC and PacBioToCA

We are aware of only one alternative program for the combined analysis of LR and SR data. The program PacBioToCA [11] also makes use of information in SR to correct errors in LR. We compared the performance of LSC with PacBioToCA (the latest version on March 13, 2012) on the same LR and SR data sets. PacBioToCA output 13,995 ecLRs, 13,980 (99.89%) of which are longer than 460 bp. Comparing with PacBioToCA, LSC has significantly higher sensitivity as it output a several-fold higher number of ecLRs in every bin of read length (Figure 5).

We divided the 62,465 LSC ecLRs with sequence identities higher or equal to 0.9 into 3 groups according to their sequence identify and read length (L) (Figure 6). Group 1 (I = 0.9665 and L = 917 bp at average) has 13,995 ecLRs, which is essentially equivalent to the reads output from PacBioToCA (Table 3). Group 2 (I = 0.9246 and L = 929 bp at average) has slightly lower identity but may still be of high quality and should not be discarded. In order to compare the qualities of Group1 and Group 2, we aligned both groups to the transcriptome and the genome by BLAT and counted how many known exon junctions can be detected respectively. From two alignments (against transcriptome and genome) of each read, the one with more known junction detections were counted. About the same numbers of true splices were detected from Group 1 and Group 2 at every bin (Table 4). Given that the two groups are similar in size, this comparison indicates that the detection from Group 2 should be of similar reliability to that from Group 1. By providing this large group of additional ecLRs, LSC has extracted a larger amount of useful information to us from the same data.

With an eight-core server (Intel(R) Xeon(R) CPUs, 2.66 GHz) with 32 G memory, LSC finished the computation within 10 hours and used about 20 G disk space to store temporary files. With the same machine, PacBioToCA took 81 hours of computation time and required a much higher amount of disk space (800∼1,000 G) for temporary files. Thus, LSC is considerably more efficient computationally. The online introduction of PacBioToCA shows that it is developed originally for the assembly of small genomes such as *E. coli*. It is perhaps not surprising that it is not competitive with LSC in the analysis of reads from the mammalian transcriptome, a task that LSC was specifically optimized for handling.

## Discussion

We have presented a method for the combined analysis of data from second generation sequencers and third generation sequencers, with the former delivering a massive (10^8^) number of accurate short reads and the latter providing a modest number (10^5^) of much longer but noisy reads. Our method yields error-corrected long reads that should be very useful for many types of downstream analyses. For example, over half of the intact gene transcripts in the human genome are shorter than 2,500 bp (Figure S1), which is close to the mean length of raw reads from the current chemistry on Pacific Biosciences’ sequencing platform. With the application of LSC, we could make full use of PacBio long reads to capture such transcripts in their intact form, and thereby to detect the corresponding gene isoform with great certainty. As an example, Figure 7 shows that an LSC ecLR (4,259 bp) covers a novel isoform of GPM6B fully. It is interesting to observe that without LSC error correction based on SRs, the raw LR cannot detect two different final 3′ exons. By detecting perfect matches of splices, 5,181 ecLRs cover 1,171 full-length gene isoforms in RefSeq annotation (7,794 ecLRs and 1,900 full-length gene isoforms in Ensembl). Besides isoform discovery in RNA-seq, LSC may also be useful in genome sequencing studies as the corrected long reads provide longer range linkage information valuable for de novo assembly of smaller genomes such as those of bacterial species.

## Supporting Information

Figure S1 The cumulative distribution functions of transcript lengths from RefSeq and Ensembl.
(TIF)

Table S1 The HC ratios of human chromosomes and the entire human genome.
(DOC)
