# Supplementary figures and images for: Improving PacBio Long Read Accuracy by Short Read Alignment
Source: PLoS One. 2012 Oct 4;7(10):e46679. doi: 10.1371/journal.pone.0046679 (PMC3464235; doi:10.1371/journal.pone.0046679)

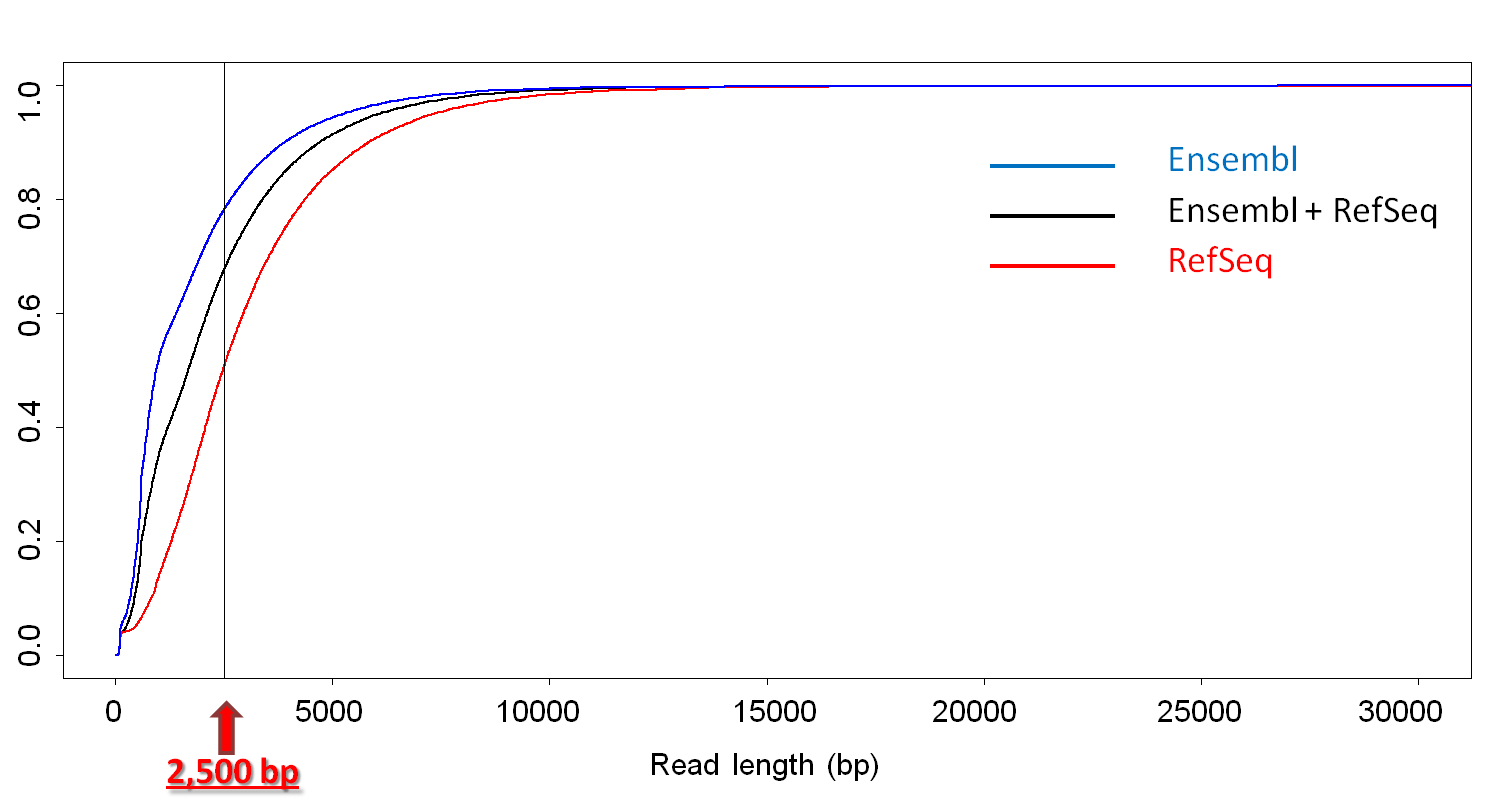

Supplement: Figure S1 — The cumulative distribution functions of transcript lengths from RefSeq and Ensembl. (TIF) [file pone.0046679.s001.tif]
