# Supplementary material for: Improving PacBio Long Read Accuracy by Short Read Alignment
Source: PLoS One. 2012 Oct 4;7(10):e46679. doi: 10.1371/journal.pone.0046679 (PMC3464235; doi:10.1371/journal.pone.0046679)
Supplement: Table S1 — The HC ratios of human chromosomes and the entire human genome. (DOC) [file pone.0046679.s002.doc]

Table S1. The HC ratios of human chromosomes and the entire human genome.

|  | **Compressed** | **Decompressed** | **Ratio** |
| --- | --- | --- | --- |
| chr10 | 92031657 | 135534747 | 0.679026 |
| chr11 | 91968840 | 135006516 | 0.681218 |
| chr12 | 91297996 | 133851895 | 0.682082 |
| chr13 | 66738266 | 115169878 | 0.579477 |
| chr14 | 61765690 | 107349540 | 0.57537 |
| chr15 | 57261765 | 102531392 | 0.55848 |
| chr16 | 55506839 | 90354753 | 0.614321 |
| chr17 | 54577460 | 81195210 | 0.672176 |
| chr18 | 52254688 | 78077248 | 0.669269 |
| chr19 | 39211196 | 59128983 | 0.663147 |
| chr1 | 157865330 | 249250621 | 0.63336 |
| chr20 | 41845983 | 63025520 | 0.663953 |
| chr21 | 24558092 | 48129895 | 0.510246 |
| chr22 | 24625094 | 51304566 | 0.479979 |
| chr2 | 166630708 | 243199373 | 0.685161 |
| chr3 | 136159148 | 198022430 | 0.687595 |
| chr4 | 130922125 | 191154276 | 0.684903 |
| chr5 | 124215714 | 180915260 | 0.686596 |
| chr6 | 116940152 | 171115067 | 0.683401 |
| chr7 | 108666793 | 159138663 | 0.682843 |
| chr8 | 100067014 | 146364022 | 0.683686 |
| chr9 | 84112895 | 141213431 | 0.595644 |
| chrM | 11767 | 16571 | 0.710096 |
| chrX | 105695362 | 155270560 | 0.680717 |
| chrY | 18023192 | 59373566 | 0.303556 |
| The whole genome | 2002953766 | 3095693983 | 0.647013 |
